# Safety risks and ethical governance of biomedical applications of synthetic biology

**DOI:** 10.3389/fbioe.2023.1292029

**Published:** 2023-10-24

**Authors:** Yakun Ou, Shengjia Guo

**Affiliations:** ^1^ School of Marxism, Huazhong University of Science and Technology, Wuhan, China; ^2^ Center for Bioethics, Huazhong University of Science and Technology, Wuhan, China

**Keywords:** synthetic biology, participant safety, biosafety risks, biosecurity risks, ethical governance, public policy

## Abstract

**Background:** In recent years, biomedicine has witnessed rapid advancements in applying synthetic biology. While these advancements have brought numerous benefits to patients, they have also given rise to a series of safety concerns.

**Methods:** This article provides a succinct overview of the current research on synthetic biology’s application in biomedicine and systematically analyzes the safety risks associated with this field. Based on this analysis, the article proposes fundamental principles for addressing these issues and presents practical recommendations for ethical governance.

**Results:** This article contends that the primary safety risks associated with the application of synthetic biology in biomedicine include participant safety, biosafety risks, and biosecurity risks. In order to effectively address these risks, it is essential to adhere to the principles of human-centeredness, non-maleficence, sustainability, and reasonable risk control. Guided by these fundamental principles and taking into account China’s specific circumstances, this article presents practical recommendations for ethical governance, which include strengthening ethical review, promoting the development and implementation of relevant policies, improving legal safeguards through top-level design, and enhancing technical capabilities for biocontainment.

**Conclusion:** As an emerging field of scientific technology, synthetic biology presents numerous safety risks and challenges in its application within biomedicine. In order to address these risks and challenges, it is imperative that appropriate measures be implemented. From a Chinese perspective, the solutions we propose serve not only to advance the domestic development of synthetic biology but also to contribute to its global progress.

## 1 Introduction

Synthetic biology is an emerging life science field in the 21st century, and there is not universally accepted definition at present. It broadly defines as a set of enabling tools allowing the modification of existing biological systems found in nature or by constructing entirely new artificial biological systems (Endy, 2005; Singh et al., 2022). One prominent strand of work in synthetic biology aims to create a range of standardized biological parts or modules that can be tacked on to bacterial chassis to produce customized biological systems (Douglas and Savulescu, 2010). It has some unique technical characteristics that distinguish synthetic biology from conventional biotechnology. The interdisciplinary nature, engineering design concept, and standardization of synthetic biology give it great capabilities, which greatly improve efficiency of designing and manufacturing life, provide robust technical support for cost-effective, large-scale, eco-friendly, and efficient pharmaceutical research and development, medical diagnostics, and clinical treatments. However, synthetic biology has made it easier to create life, greatly increased the accessibility of technology, made it accessible to many people without a background in biology, contributed to the rise of DIY Biology, and greatly increased the potential for misuse of the technology. In addition, synthetic biology also expands the threat of bioterrorism, potentially causes irreversible and devastating damage to human health and the environment, and poses more serious biosafety and biosecurity risks than conventional biotechnology. Consequently, the established developmental, operational and containment standards of conventional biotechnology are not adequate for synthetic biology applications, and new norms and standards need to be established urgently to promote the healthy development of synthetic biology.

## 2 The biomedical applications of synthetic biology

Utilizing novel biological techniques within biomedicine is not exclusive to synthetic biology. Over 3 decades ago, researchers employed genetic engineering to create an array of biopharmaceuticals, including insulin and vaccines for the human papillomavirus (Goeddel et al., 1979). The implementation of synthetic biology and its associated methodologies has further catalyzed innovation within the biomedical industry, facilitating significant advancements in pharmaceutical research, medical diagnostics, and clinical therapeutics. This has garnered the attention of a growing number of academic institutions, biotechnology firms, and pharmaceutical companies, leading to increased investment in related research endeavors.

### 2.1 The application of synthetic biology in advancing pharmaceutical research and development

The utilization of synthetic biology in pharmaceutical research and development has primarily been focused on drug discovery and vaccine development. In drug discovery, synthetic biology can aid in expanding the scale of drug production. By leveraging synthetic biology to alter the genomes of microorganisms, the process of drug production and development can become more cost-effective, efficient, and less vulnerable to environmental factors (Grinstein, 2021). For instance, opioid medicines such as morphine and codeine, which are used for treating severe pain, pain management, and palliative care (Childers et al., 2015), were previously only extractable from poppies. Despite the high market demand for opioid medicines, the growth conditions for poppies that extract and prepare these drugs are stringent and vulnerable to external factors such as climate change and pests, resulting in unstable yields. However, through the implementation of synthetic biology, a series of well-designed metabolic modules were introduced into eukaryotic yeast, enabling the production of opiate compounds through sugar fermentation (Galanie et al., 2015).

Additionally, artemisinin is a highly effective antimalarial drug. Due to the generally low artemisinin content in wild Artemisia annua plants, large-scale mass production has been challenging to achieve, resulting in an inability to meet medical demands. Researchers such as Keasling at the University of California Berkeley applied synthetic biology to microbial metabolic engineering to address this issue. He utilized low-cost industrial microorganisms to ferment and produce artemisinin (Ro et al., 2006). This artificial synthesis method overcomes the disadvantages of low yields and long extraction cycles associated with wild Artemisia annua plants. It provides a more efficient and environmentally friendly means of production.

The utilization of synthetic biology in vaccine research and development has the potential to expedite the vaccine development process while simultaneously providing a theoretical foundation and practical support for the prevention and control of diseases. For instance, in 2018, Chinese researchers employed synthetic biology to develop a novel vaccine for the Zika virus. This vaccine not only boasts a reduced production time but also exhibits enhanced safety, efficacy, and immunogenicity (Li et al., 2018). Synthetic biology also played a crucial role in developing the COVID-19 vaccine. In May 2020, Swiss researchers synthesized the novel coronavirus and other analogous RNA viruses via genome-wide synthesis. This comprehensive synthesis approach enables the production or modification of a substantial quantity of live SARS-CoV-2 viruses within a week for utilization by medical and research institutions, thereby accelerating the development of COVID-19 vaccines and facilitating a rapid response to the pandemic (Thi Nhu Thao et al., 2020).

### 2.2 The application of synthetic biology in medical diagnostics

In medical diagnostics, synthetic biology facilitates the dynamic monitoring of human health and the precise evaluation of disease severity through modifying the genomes of cells or microorganisms, imbuing them with the capacity to detect abnormal cells and identify lesions within the body. For example, the CRISPR-Cas9 system can be employed to construct biological circuits within cells that specifically recognize key protein molecules in intracellular cancer signaling pathways, providing more accurate determinations for locating cancer cells and assessing disease progression (Liu et al., 2014). Researchers at Columbia University in the United States have utilized CRISPR technology to modify *Escherichia coli*, enabling it to record and monitor changes in the human digestive tract (Sheth et al., 2017). This has yielded unprecedented insights into previously unobservable phenomena and can even be applied to environmental monitoring, ecology, and microbiology.

Synthetic biology can also be employed to detect allergic and inflammatory responses. Allergic diseases are intricate chronic conditions wherein allergens constitute the fundamental cause of allergic and inflammatory reactions (Aldakheel, 2021). Consequently, detecting and screening allergens at their source is critical in medical diagnostics. In diagnosing allergic diseases, advancements in synthetic biology have expedited the development of cell-based biosensors for clinical applications. By sensing biomarkers associated with inflammation, immunity, and metabolic disorders via biosensors, novel diagnostic and treatment systems can be devised and established (Inda et al., 2019). Synthetic biology can further facilitate the technological transformation and upgrading of allergy detection products. For instance, an engineered mammalian cell detection system can be employed for allergy testing during new drug development (Zhao et al., 2023). The efficiency of new drug development can be significantly enhanced by conducting high-throughput screening of blood samples from high-risk allergy patients.

### 2.3 The application of synthetic biology in clinical treatment

The clinical treatment represents one of the most significant applications of synthetic biology within the field of biomedicine and constitutes a primary objective of synthetic biology development. Clinical treatments encompassing synthetic biology include gene therapy, cell immunotherapy, and engineered therapeutic bacteria or viruses (Caliendo et al., 2019; Chakravarti and Wong, 2015). Gene therapy is among the most advanced application domains of synthetic biology. On 27 November 2020, SyngenTech announced that its world-first gene therapy product SynOV1.1, developed utilizing synthetic biology, had received clinical trial authorization from the US FDA and had undergone phase I and II clinical studies at the world’s largest private cancer research center—Memorial Sloan Kettering Cancer Center, which can be employed in the treatment of liver cancer (Liu et al., 2021). Cell immunotherapy is also an application domain of synthetic biology, with Chimeric antigen receptor (CAR)-T therapy being the most representative. CAR is a synthetically engineered receptor designed to redirect lymphocytes (most commonly T cells) to recognize and eliminate cells expressing specific target antigens (Sterner and Sterner, 2021). It exhibits characteristics such as precision, high efficiency, and rapidity and has demonstrated favorable outcomes in treating leukemia and malignant lymphoma, with high expectations for its potential to cure cancer (Zhang et al., 2023). In addition to modifying cells, synthetic biology can also be applied to modify bacteria and viruses. For instance, attenuated *Salmonella* modified via synthetic biotechnology can effectively reduce tumor volume, delay tumor growth, and enhance the capacity to kill tumor cells, offering hope and a new dawn to tens of millions of tumor patients worldwide (Chen et al., 2021).

Synthetic biology possesses the potential to address the crisis of antibiotic resistance. In 2022, a research team at Rockefeller University in the United States synthesized a novel antibiotic, Cilagicin, predicated on a computational model of bacterial gene products. This antibiotic has demonstrated favorable outcomes in mice and, owing to its innovative mechanism of targeting lethal pathogens, exhibits diminished resistance compared to traditional antibiotics (Wang et al., 2022).

## 3 An analysis of the safety risks pertaining to the application of synthetic biology within biomedicine

The field of biomedicine presents a vast array of opportunities for applying synthetic biology. However, these opportunities are accompanied by a series of safety risks that must be carefully considered. Clinical trials involving drugs, vaccines, diagnostics, and treatments have the potential to cause harm to Subjects. Furthermore, the unintentional release of synthetic organisms may result in a range of biosafety concerns, including health risks to laboratory personnel and threats to the safety of surrounding communities and the ecological environment. Additionally, the malicious use or abuse of synthetic organisms may give rise to biosecurity issues.

### 3.1 Issues pertaining to the safety of subjects

In order to ascertain the safety and effectiveness of drugs, vaccines, diagnostic methods, and treatment modalities, it is imperative to conduct clinical trials. Given the distinct nature of synthetic biology and biotechnology, it can recreate known pathogenic viruses, make biochemicals via *in situ* synthesis, make existing bacteria more dangerous (National Academies of Sciences, Engineering, and Medicine, 2018), subjects participating in the trial may be exposed to health risks such as allergies, toxicity, pathogenicity, antibiotic resistance, and even carcinogenicity. Consequently, the safety of subjects is an inescapable concern.

First and foremost, clinical trials employing synthetic biology may jeopardize the safety of subjects due to factors such as inadequate experimental design and non-compliant procedures. In October 2022, the sole global participant in a Duchenne muscular dystrophy (DMD) trial tragically passed away following the administration of CRISPR gene editing therapy via an Adeno-associated virus (AAV) vector (Dongsheng, 2023; Philippidis, 2022). The precise cause of the subject’s demise remains under investigation, with some researchers positing that it was precipitated by a potent immune response to the high dosage of the AAV vector (Lek et al., 2023).

Negligent clinical trials not only inflict grave harm or even death upon subjects but also impede progress in related research. The notorious Gelsinger trial exemplifies how the death of a subject can result in a regression in gene therapy research. Jesse Gelsinger, an 18-year-old afflicted with a rare condition known as Ornithine transcarbamylase deficiency (OTCD), perished after undergoing experimental gene therapy spearheaded by James Wilson’s laboratory at the University of Pennsylvania (Marshall, 1999). A subsequent inquiry by the FDA uncovered numerous instances of malpractice in the University of Pennsylvania’s OTCD gene therapy clinical trial, which bore an undeniable responsibility for Gelsinger’s untimely death (Marshal, 2000). Presently, synthetic biology is also being applied to gene therapy and even more intricate treatment modalities, such as cellular immunotherapy and targeted therapy using engineered bacteria. These trials are inherently fraught with uncertainty and necessitate more rigorous scientific design with paramount emphasis on subject safety.

Secondly, the development of COVID-19 vaccines utilizing synthetic biology and biotechnology also raises safety and ethical issues during human trials. DNA and RNA vaccines and adenovirus vector vaccines entail synthesizing viral genes and modifying nucleic acid sequences (Kitney et al., 2021). Synthetic biology and biotechnology have been instrumental in expediting the vaccine development process and enhancing the immunogenicity and breadth of vaccines. Nonetheless, even if we can rapidly comprehend the characteristics of the virus or design its sequence using synthetic biology and biotechnology, our grasp of the interplay between the virus and the human immune system and which type of immune response is optimal for eliciting enduring effective immunity remains limited (Zhaoling et al., 2023). The safety and efficacy of vaccines necessitate protracted clinical trials and observation. However, to expedite testing of the effectiveness of COVID-19 vaccines developed via different technological pathways, some countries have initiated Human Challenge Trials (HCT), wherein a cohort of healthy volunteers are administered different test vaccines before being deliberately exposed to the virus to assess the vaccine’s immune effect, to accelerate clinical data collection and reduce the time required for vaccine approval testing (Yueyue and Yali, 2021).

In 2021, the United Kingdom became the first nation globally to conduct a human challenge trial for COVID-19, wherein subjects were initially inoculated with different COVID-19 test vaccines before being infected with a “challenge” dose of COVID-19 in a controlled setting to evaluate the vaccine’s immune effect (Killingley et al., 2022; Kirby, 2020). HCT remains a contentious testing methodology to this day. Subjects are required to undergo isolation for several days during the entire trial process. Although researchers meticulously monitor the entire trial process and medical personnel are on hand to provide treatment to volunteers if required, it is still impossible to fully guarantee subject safety (Williams et al., 2022).

### 3.2 Biosafety issues

Apart from the safety of subjects, the utilization of synthetic biology in biomedicine also presents biosafety challenges. The World Health Organization delineates “Biosafety” in its Laboratory Biosafety Manual as “containment principles, technologies and practices that are implemented to prevent unintentional exposure to biological agents or their inadvertent release (World Health Organization, 2004).” Synthetic biology has tremendous capabilities and has greatly facilitated the development of drugs and vaccines. But at the same time, it can also produce more toxic, infectious, and dangerous pathogens. Any improper operation or accidental contact may imperil laboratory personnel, neighboring communities, and the ecological environment.

Concerns derive from the capabilities of synthetic biology can pose inherent harm. Imminent concerns include re-creating known pathogenic viruses, making existing bacteria more dangerous, and making harmful biochemicals via *in situ* synthesis. These capabilities are based on knowledge that are readily available to a wide range of participants. Medium concerns include manufacturing chemicals or biochemicals by exploiting natural metabolic pathways and the use of synthetic biology to make existing viruses more dangerous, also include manufacturing chemicals or biochemicals by creating novel metabolic pathways, efforts to modify the human microbiome to cause harm, efforts to modify the human immune system, and efforts to modify the human genome. These capabilities involve more constraints and may be limited by factors related to biology and skill. Long-term concerns include re-creating known pathogenic bacteria and creating new pathogens, these capabilities involve implementation challenges. The use of human gene drives requires a minimal level of concern, as it is impractical to rely on sexual reproduction over several generations to spread harmful traits. (National Academies of Sciences, Engineering, and Medicine, 2018).

In summary, synthetic biology has the capabilities to produce more dangerous pathogens or organisms, which may exert deleterious effects on human health and the environment if accidentally released. Based on an analysis of approximately 200 articles, Joel Hewett et al. determined that the human health risks posed by synthetic biology primarily encompass: allergies; antibiotic resistance; carcinogens; and pathogenicity or toxicity. The environmental risks posed by synthetic biology primarily encompass: change or depletion of the environment; competition with native species; horizontal gene transfer; and pathogenicity or toxicity (Hewett et al., 2016). The impact of synthetic organisms or pathogens is also contingent upon factors such as the species which is designed, the nature of the change, the site of release, and the characteristics of genetic modification, particularly when alterations transpire in the toxicity, infectivity, adaptability, and host interaction mechanisms of pathogenic organisms (Bohua et al., 2023). In certain instances, once synthetic organisms or pathogens are inadvertently released, their harm may be amplified through ecological cycles, ultimately surpassing the carrying capacity of ecosystems and inflicting greater collateral damage.

In response to these concerns and risks, the National Academies of Sciences, Engineering, and Medicine have proposed mitigation options that include: 1)Relevant government departments should continue exploring strategies to address chemical and biological defense threats. 2)Relevant government agencies should assess national military and civilian infrastructure to provide information for population-based surveillance, identification and communication of natural and purposeful health threats. 3)The government should work with the scientific community to develop strategies to manage emerging risks, rather than relying solely on current agent-based lists and access control approaches. (National Academies of Sciences, Engineering, and Medicine, 2018).These measures have been effective, but the potential problems posed by synthetic biology will remain a challenge for scientists and national defenses, and continuous efforts are also needed to promote scientific and technological progress while reducing risks.

### 3.3 Biosecurity issues

Apart from biosafety concerns, synthetic biology also engenders biosecurity issues. The Laboratory Biosafety Manual delineates Biosecurity as “Principles, technologies and practices that are implemented for the protection, control and accountability of biological materials and/or the equipment, skills and data related to their handling. Biosecurity aims to prevent their unauthorized access, loss, theft, misuse, diversion or release (World Health Organization, 2004).” If synthetic organisms or pathogens are employed to conduct biological warfare or bioterrorism, the extent, duration, and magnitude of harm would be unfathomable. Moreover, the emergence of DIY biology has further exacerbated the biosecurity challenges posed by synthetic biology in the biomedical domain.

#### 3.3.1 Bioterrorism

“Bioterrorism” entails deliberately employing microorganisms or toxins as infectious agents to induce disease or death in humans, animals, or plants and intentionally engender fear among populations (Bossi et al., 2006). Unlike other forms of terrorism, such as nuclear weapons, bioterrorism can more readily inflict widespread destruction globally and is thus dubbed the “poor man’s nuclear weapon (Poor Toulabi, 2023).” The advancement of synthetic biology has further facilitated bioterrorism by augmenting the capacity of malevolent actors to generate injurious biological agents with diminished resources.

For scientific research purposes, researchers may utilize synthetic biology to “resurrect” natural pathogens or modify extant pathogens (Noyce et al., 2018), or amalgamate genetic material from multiple pathogens to explore novel avenues for vaccine development (Sanders et al., 2016). However, once biological terrorists exploit these modified pathogens, they present incalculable hazards. For instance, in October 2022, researchers at Boston University announced that they had created a novel strain of the COVID-19 virus by fusing the spike protein of the Omicron variant with the strain infecting the first confirmed COVID-19 patient in the United States, and this synthetic strain is five times more infectious than the Omicron variant and has a mortality rate of up to 80% (Chen et al., 2022). In reaction, David Livermore, a professor of microbiology at the University of East Anglia in the UK, opined that such virus modification experiments are exceedingly unwise and undesirable; Professor Shmuel Shapira, Israel’s chief scientist, contended that this constitutes “playing with fire” and should be categorically prohibited (Tilley et al., 2023). They both concur that if this highly perilous synthetic virus were to leak or be malevolently exploited, it would furnish an opportunity for bioterrorism and could wreak catastrophic havoc worldwide.

Additionally, owing to the open access mechanism of synthetic biology and its development tenets of engineering, informatization, and technical simplification, while broadening the accessibility of technology, it also heightens the risk of malevolent exploitation by biological terrorists (Melin, 2021; Trump et al., 2020). Publicly accessible gene sequence information and synthetic biotechnologies furnish a “blueprint” and technical tools for developing bioterrorism and biological weapons, rendering it less expensive and more convenient to fabricate pathogens using synthetic biology.

#### 3.3.2 DIY biology

The assembly of biological components to create new drug reagents has been simplified and accelerated through the use of standardized and engineered methods. This has led to the emergence of a large number of DIY biology practitioners, including biohackers and garage biologists (Ikemoto, 2017). The aim of DIY biology is to break down the barriers imposed by traditional laboratories, disseminate knowledge about synthetic biology, promote open access and sharing of resources, and provide opportunities for everyone to engage in scientific practice (Kuznetsov et al., 2015). Most practitioners are not motivated by profit but are dedicated to using synthetic biology to develop affordable and convenient biotechnology equipment that offers alternative solutions to medical challenges faced by humanity and benefits underprivileged or underdeveloped communities. For instance, Dutch DIY biologist Bruins and his colleagues used simple devices such as hairdryer heaters, shoeboxes, and electronic products to create “Amplino”—a low-cost, high-sensitivity mobile malaria test kit (Landrain et al., 2013). This reagent is more affordable, accessible, and user-friendly than traditional diagnostic tools. Individuals can test for malaria in their homes, thereby advancing the field of malaria test and other disease detection.

DIY biology has bridged the gap between synthetic biology and the general public, attracting many interdisciplinary practitioners to research synthetic biology and invigorating technological development with creativity and dynamism. However, caution must be exercised to mitigate the risks associated with lowering technical barriers. Most DIY practitioners are amateurs who lack formal training in laboratory safety and systematic knowledge of scientific theory. The absence of specialized laws and regulations, as well as departmental oversight, can result in the misuse of technology. Zosiah Zayner, founder of ODIN in California, United States, is a proponent of DIY biology who advocates for making gene editing accessible to more people (Guerrini et al., 2019). This has raised concerns among scholars who argue that the use of gene editing outside laboratories should be restricted (West and Gronvall, 2020). These concerns are not unfounded, as DIY biology practitioners conduct experiments based on personal interests without adhering to standard operating procedures or being able to predict and ensure experimental safety. This could potentially result in biosafety and biosecurity risks.

## 4 Ethical considerations in the application of synthetic biology within the biomedical field

In order to address the safety risks associated with the application of synthetic biology within the biomedical field, it is imperative to establish appropriate countermeasures. The development of these countermeasures must be grounded in and guided by fundamental ethical principles. Accordingly, we propose four fundamental principles to govern the use of synthetic biology in this domain: human-centeredness, non-maleficence, sustainability, and reasonable risk control. These principles are intended to foster the responsible and healthy advancement of synthetic biology within the biomedical field.

### 4.1 The principle of human-centeredness

The principle of human-centeredness underscores the importance of valuing and respecting human life, addressing human needs, health, and wellbeing, and advocating for the application of science and technology to enhance human welfare. This principle has long been a fundamental tenet of human society. In China, the “Great Declaration I” in the Book of History (尚书·泰誓上) states that “Heaven and Earth are parents of all creatures, and of those, Man is the most highly accomplished (Li, 2022),” representing one of the earliest written affirmations of the value of humanity. Similarly, humanistic traditions also exist in other cultures; for example, the ancient Greek philosopher Protagoras famously declared that “Man is the measure of all things (Kattsoff, 1953).” In summary, the principle of human-centeredness is a crucial ethical principle that is essential for understanding the relationship between humans and nature and provides important guidance for the safe and ethical governance of synthetic biology within the biomedical field.

In applying synthetic biology within the biomedical field, it is essential to adhere to the principle of human-centeredness by respecting life, safeguarding the safety and rights of patients and research participants, honoring individual autonomy, upholding human dignity, and ensuring informed consent. Many guidelines and regulations governing clinical trials reflect this principle. For example, China’s Good Clinical Practice (GCP) stipulates that the rights and safety of research participants are primary considerations that take precedence over scientific and societal benefits (Jiyin, 2021). The Declaration of Helsinki emphasizes that during human experimentation, researchers must ensure research participants’ physical, psychological, and social wellbeing (World Medical Association, 2013). In 2023, China’s Measures for the Ethical Review of Life Science and Biomedical Research Involving Humans provide detailed provisions for protecting the privacy rights, informed consent rights, and compensation rights of research participants, requiring researchers to protect the rights of participants in clinical trials by closely monitoring their medication use, health status, and changes in clinical data, and ethics review committees are responsible for reviewing whether research participants have been treated unfairly and for promptly addressing their concerns (National Health Commission, 2023). Adherence to the principle of human-centeredness in applying synthetic biology within the biomedical field also requires that synthetic biology and biotechnologies always strive to enhance human welfare as their ultimate goal. Ethical considerations must be integrated throughout the entire technology development process to promote benevolent technological advancement that amplifies human goodness and achieves moral development by using technology to address societal challenges and ensure that technological achievements benefit humanity.

### 4.2 The principle of non-maleficence

The principle of non-maleficence is the most fundamental and bottom-line ethical principle in bioethics. In today’s morally diverse world, this principle serves as a “global ethic” or “universal ethic” that is widely recognized and applied worldwide (Linklater, 2006). The principle of non-maleficence does not require the complete avoidance of harm; instead, it acknowledges that the development of any technology inevitably brings some degree of harm and necessitates weighing potential harms to choose the lesser harm. For example, in human challenge trials for COVID-19 vaccines, participants who received different types of vaccines developed varying degrees of COVID-19 symptoms. Does this violate the principle of non-maleficence? The answer is no. We consider that clinical human trials are an essential stage in the development of COVID-19 vaccines and play a crucial role in testing their safety and efficacy. As long as relevant laws, regulations, and ethical norms are followed, and informed consent from participants is obtained, the application of HCT is ethically reasonable. It is commendable for volunteers to sacrifice their own health for the benefit of humanity when they are fully aware and willing of the risks of the experiment. Therefore, although HCT may cause some harm to participants, it still applies to “principle of non-maleficence”. The principle of non-maleficence is not a principle of no harm but rather a principle of minimal harm, reasonable harm, or morally permissible harm (Jianbing and Chuanzhong, 2007).

### 4.3 The principle of sustainability

The principle of sustainability is a goal-oriented principle that aims to achieve long-term harmony between humans and nature by meeting the needs of the present generation without compromising the ability of future generations to meet their own needs (Munthe et al., 2021). This intergenerational ethical principle requires the present generation to respect future generations’ rights to life and development and not deprive them of their rights simply because they do not yet exist or have no voice. Sustainability is closely related to sustainable development, but the two concepts are distinct. Sustainability is a broader concept, while sustainable development focuses primarily on human welfare (Harrington, 2016). Additionally, the two concepts have different emphases: sustainability emphasizes the long-term nature of goals, while sustainable development focuses on the processes and pathways for achieving these goals. Generally speaking, the principle of sustainability encompasses ecological, economic, and social sustainability, all interconnected and inseparable (Berg, 2020). Among these, ecological sustainability is considered the most important and directly affects the other two types of sustainability.

The research and application of synthetic biology in the biomedical field may have irreversible and severe impacts on the ecological environment. Therefore, it is essential to adhere to the principle of sustainability to avoid sacrificing the ecological environment and the welfare of future generations for technological advancement and to ensure the sustainable development of ecology, economy, and society. To achieve these goals, several action principles must be followed:

Firstly, in terms of the relationship between humans and nature, the principle of sustainability requires researchers to follow the precautionary principle by proactively taking preventive measures to reduce or avoid risks to the natural environment when harm is uncertain; Secondly, the principle of sustainability requires researchers to adhere to the prudence principle. Synthetic biology is highly complex and uncertain; researchers must adhere to the prudence principle as a core behavioral norm and be responsible for themselves, future generations, and the ecological environment.

Thirdly, regarding the relationship between humans and society, the principle of sustainability requires providing maximum compensation and support to vulnerable groups; seeking public understanding and trust; strengthening unity and cooperation; involving all stakeholders in research; enhancing policy transparency; etc.

### 4.4 The principle of reasonable risk control

In order to address the biosafety and biosecurity concerns associated with synthetic biology in the biomedical field, it is essential to adhere to the principle of reasonable risk control. This principle mandates that managers implement measures to reduce or eliminate the likelihood of risk occurrence or to keep risks within an acceptable range to prevent incurring unbearable losses (Aven, 2016). In March 2022, the General Office of the Central Committee of the Communist Party of China and the General Office of the State Council issued the Opinions on Strengthening the Governance of Science and Technology Ethics, which proposed five principles of science and technology ethics, including the principle of reasonable risk control. This principle stipulates that scientific activities must objectively evaluate and prudently address the uncertainty and risks associated with technology and its application; Efforts must be made to avoid and prevent potential risks, prevent the misuse or abuse of scientific achievements, and avoid endangering social security, public security, biosafety, and ecological safety (General Office of the Central Committee of the Communist Party of China and General Office of the State Council, 2022). The introduction of the principle of reasonable risk control is beneficial in addressing ethical challenges posed by emerging technologies such as synthetic biology and promoting the healthy development of science and technology.

Specifically, the application of synthetic biology in biomedicine must adhere to the natures of effectiveness, advancement, whole process, initiative, and systematicity. Firstly, the effectiveness requires scientists to effectively identify potential hazards in pharmaceutical research and development as well as disease treatment processes. Subsequently, operable management measures must be formulated for identified hazards to improve risk control effectiveness. Secondly, the advancement necessitates developing or introducing advanced risk control technologies and effectively utilizing them in conjunction with China’s synthetic biology industry’s characteristics. Then, the whole process mandates strict control of risks at various stages of experiments related to synthetic biology through independent risk assessment and dynamic supervision throughout the whole experiment process. Additionally, research applications must undergo strict scrutiny. Furthermore, adherence to proactive control and prior control thinking is required by the nature of initiative. In response to changing environmental conditions and emerging new situations and problems, timely response measures must be taken, and response plans adjusted. Finally, risk control is a highly systematic and comprehensive task. Especially in interdisciplinary fields such as synthetic biology where risks have complex origins and far-reaching consequences, it is necessary to formulate more risk management measures.

## 5 Recommendations for ethical governance of biomedical applications of synthetic biology

In order to address safety concerns associated with the application of synthetic biology within the biomedical field, it is necessary to develop practical governance measures guided by the aforementioned fundamental principles. We believe that efforts can be made in several areas, including strengthening ethical review, promoting the development and implementation of relevant policies, improving legal safeguards through top-level design, and enhancing technical capabilities for biocontainment.

### 5.1 Strengthening ethical review

The widespread application of synthetic biology within the biomedical field has led to a sharp increase in safety risks, necessitating the development of new legal and ethical regulations and the strengthening of ethical review to ensure the safety of research participants, biosafety, biosecurity, and the prevention of exploitation by bioterrorists. Currently, China has issued regulations such as GCP (National Health Commission, 2020), Measures for the Ethical Review of Life Science and Biomedical Research Involving Humans (National Health Commission, 2023), and Guiding Principles for Ethical Review of Drug Clinical Trials (EOCJRDU, 2010), providing institutional safeguards for strengthening ethical review of clinical trials, regulating the work of ethics review committees, and ensuring compliance with scientific and ethical requirements. However, the research and application of synthetic biology within the biomedical field have disrupted traditional ethical review paradigms for clinical trials. Its enormous technological power and influence pose a serious threat to the safety of research participants and present unprecedented challenges to biosafety and biosecurity. The existing ethical review paradigm can no longer meet the development needs of synthetic biology within the biomedical field, and there is an urgent need within academia to establish a new ethical review paradigm to ensure its healthy development.

The establishment of new ethical review paradigm depends on ethics committees. At present, ethics committees are composed mainly of biologists, medical scientists and other scientists, many of whom lack the ethical literacy, and only consider what can be done, rather than what should be done, resulting in a lack of rationality in ethical review.In this regard, we call for the participation of humanities and social scientists such as bioethicists, lawyers and sociologists to join the ethics committee, and invite the participation of stakeholders such as public representatives and religious figures. In addition to disciplinary background, the composition of the ethics committee shall take into account factors such as gender, age, education, ethnicity and geographical distribution of the members, and they shall be independent of the research/experimental unit in conducting reviews, making recommendations and making decisions. Ethics committees should review the design and implementation of research plans; the risks and benefits of trials; the recruitment and informed consent of research participants; their safety and privacy; and research involving vulnerable groups. The ethics committee should also develop standard operating procedures and systems for biotechnology to ensure consistency and standardization in ethical review work.

Additionally, the ethics committee should regularly provide professional training to researchers to raise their awareness of safety and social responsibility. As synthetic biology develops rapidly, ethics committees should continuously improve their organizational management and institutional development in response to technological needs, fulfill their responsibilities to protect the safety, dignity, and rights of research participants, enhance public support for and trust in the application of synthetic biology, and promote it scientific and healthy development within the field of biomedicine.

### 5.2 Promoting the development and implementation of relevant policies

The State Council should coordinate with institutions such as the China’s Center for Disease Control and Prevention, the National Health Commission, the Ministry of Agriculture and Rural Affairs, and the Ministry of Ecology and Environment to establish a safety review system for joint decision-making on safety issues related to synthetic biology. The review panel should follow safety, efficacy, and economy in conducting risk assessments of synthetic biology programs and classify them according to risk level and application type (unrestricted use, restricted use, and special use), with a focus on regulating dangerous target experiments such as synthetic viruses and bacteria. The government should also establish and improve monitoring mechanisms to regularly inspect laboratory safety management facilities and systems; supervise laboratory personnel to ensure compliance with regulations and equipment maintenance; inspect the transportation and storage of hazardous reagents; and prevent accidental harm due to negligence. In addition, the government should strictly regulate the order services of synthetic biology companies to prevent malicious exploitation by criminals.

These policies are binding on professional organizations such as research institutions and enterprises but have limited effect on private research institutions and amateur enthusiasts outside institutional arrangements. To address this issue, the government can establish formal, open community laboratories to provide DIY biology practitioners with regular research venues and regulate their research behaviour. The government can implement a registration management system in community laboratories to protect DIY biology practitioners’ legitimate rights and interests while clarifying responsibilities and scope and urging them to fulfill their laboratory safety responsibilities. In addition, the government should actively guide DIY biology practitioners to establish informal standards and regulations, encourage them to develop a sense of responsibility, improve their self-governance capabilities, and guide the enormous technological potential of DIY biology groups toward legal paths that contribute to China’s high-tech development.

Research in synthetic biology is closely related to the public interest, and the public has the right to be informed about research results, hold researchers accountable, and exercise oversight. The government should promote public communication and encourage public participation in relevant discussions, reviews, management, and decision-making. The government can establish various effective communication channels such as setting up dedicated communication departments (e.g., Synthetic Biology Consultation Office), dedicated communication time slots (e.g., regular meetings), or more convenient communication websites, mailboxes, public accounts, etc., to solicit public opinions on sensitive issues such as synthetic viruses and bacteria and listen to public voices.

### 5.3 Improving legal safeguards through top-level design

Reliance on ethical principles and guidelines alone is insufficient for governance in the application of synthetic biology within biomedicine; legal safeguards are also necessary. China has established a foundation in biosafety and biosecurity legislation, such as the Biosecurity Law implemented in 2021. This law stipulates strengthening safety management for biotechnology research, development, and application activities. Relevant activities must comply with ethical principles and are prohibited from endangering public people, such as endangering public health, damaging biological resources, or destroying ecosystems and biodiversity (Pandi W et al., 2021). The law also outlines measures to prevent bioterrorism and bioweapon threats, including prohibiting the development, manufacture, acquisition, storage, possession, and use of bioweapons. It also requires formulating a special list of organisms, biological toxins, equipment, or technologies that can be used for bioterrorism activities or manufacturing bioweapons and taking measures to prevent spreading (Haiyou, 2020).

China’s Criminal Law regulates illegal and criminal acts that endanger public safety and engage in bioterrorism. It includes explicit provisions for crimes such as the illegal manufacture, sale, transportation, and storage of dangerous substances; release of toxic, radioactive or infectious disease pathogens; organization, leadership, or participation in terrorist organizations; and assistance to terrorist activities (People’s Republic of China, 2020). China’s Counter-Terrorism Law requires strict supervision and management of infectious disease pathogens to prevent their spread or entry into illegal channels. It stipulates that, in the event of theft, robbery, or loss of infectious disease pathogens, necessary control measures must be taken immediately and reported to the public security organs and competent authorities (People’s Republic of China, 2018).

Despite China’s legal foundation for biosafety and biosecurity, there remain issues such as incomplete content and lack of punitive measures. In particular, there is still a legal, regulatory gap in synthetic biology research in the biomedical field. For example, the impact on the ecological environment of synthetic organisms or pathogens accidentally released during research has not yet been included in the scope of legal regulation. Some raw materials, such as oligonucleotides, are not included in the special list. Moreover, synthetic biology is rapidly developing. Biological toxins and equipment that can be used to launch bioterrorism or manufacture bioweapons are constantly changing. Legislative bodies should timely amend and follow up technical lists to improve legal norms related to synthetic organisms and pathogens. Strengthen safety management of pathogenic organism laboratories. Clarify new standards and requirements for synthesizing bacteria, viruses, and other pathogens to prevent them from being used to manufacture bioweapons or for terrorist purposes. In addition to strengthening biosafety and biosecurity legislation, China must promote biotechnology innovation and clarify the boundaries between technology safety and innovation, not to restrict technology development and produce a “chilling effect.” In summary, legislative bodies should establish a sound normative document for synthetic biology safety management so that synthetic biology research applications in various fields have laws to follow and must follow laws.

### 5.4 Enhancing technical capabilities for biocontainment

In order to minimize the risks associated with the application of synthetic biology in the biomedical field, it is insufficient to rely solely on external regulatory mechanisms through ethics, policy, and law. This is because accurately predicting the risks of a new product, particularly synthetic biological products that have never existed before, is challenging. It is impossible to determine their impact on humans or the ecological environment based on experience. As a result, it is necessary to design reliable internal biocontainment measures to ensure that synthetic organisms do not cause harm even if unintentionally released or maliciously used, thereby eliminating adverse effects at their source.

Common biocontainment strategies include auxotrophic organisms, toxin-antitoxin pairs, CRISPR-based “kill switches,” and xeno-nucleic acids (XNAs) (Wright et al., 2013). These methods and mechanisms can be applied to whole processes involving the development of synthetic organisms. Through these safety measures, it is possible to prevent synthetic organisms from surviving or dying under natural conditions, effectively preventing their spread to experimenters, people outside the laboratory, and the environment. Furthermore, these safety measures should be continuously updated and improved as technology advances.

## 6 Conclusion

The research and application of synthetic biology in the biomedical field can potentially address significant public health and hygiene issues. However, as an emerging science and technology, synthetic biology faces numerous challenges, including interdisciplinary intersections, technological innovations, and unknown risks. Its application in the biomedical field is particularly complex and uncertain, posing unprecedented threats to subject safety, biosecurity, and biosecurity. In response, China must establish related laws and regulations guided by fundamental ethical principles, implement suitable ethical review mechanisms, and clarify various departments and researchers’ technical and ethical responsibilities during research and development. This will diminish or eliminate risks associated with synthetic biology at different stages.

Policy guidance should also be promoted to improve relevant management systems and operating procedures. This will regulate the production and use of synthetic biology through institutional norms and prevent biosafety and biosecurity risks. Additionally, legals and regulations related to synthetic pathogens should be refined and severely punish malicious acts such as bioterrorism. The level of biocontainment must also be improved to minimize safety risks. These measures should be revised in a timely manner as synthetic biology advances. Only then can we better regulate the research and application of synthetic biology in the biomedical field and guide its positive development.
